# Third BNT162b2 mRNA SARS-CoV-2 Vaccine Dose Significantly Enhances Immunogenicity in Recipients of Allogeneic Hematopoietic Stem Cell Transplantation

**DOI:** 10.3390/vaccines11040775

**Published:** 2023-03-31

**Authors:** Israel Henig, Jonathan Isenberg, Dana Yehudai-Ofir, Ronit Leiba, Shimrit Ringelstein-Harlev, Ron Ram, Batia Avni, Odelia Amit, Sigal Grisariu, Tehila Azoulay, Ilana Slouzkey, Tsila Zuckerman

**Affiliations:** 1 Department of Hematology and Bone Marrow Transplantation, Rambam Health Care Campus, Haifa 3109601, Israel; 2 Ruth and Bruce Rappaport Faculty of Medicine, Technion, Haifa 3200003, Israel; 3 Department of Statistics, Rambam Health Care Campus, Haifa 3109601, Israel; 4 Bone Marrow Transplantation Unit, Sourasky Medical Center, Tel Aviv 6423906, Israel; 5 Sackler Faculty of Medicine, Tel Aviv University, Tel Aviv 6997801, Israel; 6 Department of Bone Marrow Transplantation and Cancer Immunotherapy, Hadassah Medical Center, Jerusalem 9112001, Israel; 7 Faculty of Medicine, Hebrew University of Jerusalem, Jerusalem 9190401, Israel; 8 Hematology Laboratory, Rambam Health Care Campus, Haifa 3109601, Israel

**Keywords:** SARS-CoV-2, BNT162b2, vaccine, allogeneic hematopoietic stem cell transplantation, immunogenicity, cellular response

## Abstract

COVID-19-related mortality among hematopoietic stem cell transplantation (HSCT) recipients in the pre-vaccine era ranged between 22 and 33%. The Pfizer/BioNTech BNT162b2 vaccine demonstrated significant immunogenicity and efficacy in the healthy population; however, its long-term effects on allogeneic HSCT recipients remained unclear. Our study longitudinally evaluated humoral and cellular responses to the BNT162b2 vaccine in adult allogeneic HSCT patients. A positive response was defined as antibody titers ≥ 150 AU/mL post-second vaccination. Among 77 included patients, 51 (66.2%) responded to vaccination. Response-associated factors were female gender, recent anti-CD20 therapy, and a longer interval between transplant and vaccination. Response rates reached 83.7% in patients vaccinated >12 months post-transplant. At 6 months post-second vaccination, antibody titers dropped, but were significantly increased with the booster dose. Moreover, 43% (6/14) of non-responders to the second vaccination acquired sufficient antibody titers after booster administration, resulting in an overall response rate of 79.5% for the entire cohort. The BNT162b2 vaccine was effective in allogeneic transplant recipients. Although antibody titers decreased with time, the third vaccination led to their significant elevation, with 93% of third-dose responders maintaining titers above 150 AU/mL at 3 months post-administration.

## 1. Introduction

Severe acute respiratory syndrome coronavirus 2 (SARS-CoV-2), the cause of the COVID-19 pandemic and exerting a powerful adverse impact on the health of the general population, is associated with a significantly increased mortality in cancer patients, particularly those with hematological malignancies [1,2,3]. 

Among the recipients of hematopoietic stem cell transplantation (HSCT) with COVID-19, the reported mortality rate assessed less than one year after the pandemic outbreak, ranged between 22% and 32% after the allogeneic HSCT and 28–33% after the autologous HSCT [4,5]. This would add an excessive mortality risk on top of a 3-year transplant-related mortality rate of 20% for the allogeneic transplant [6] and 5% for the autologous transplant recipients [7].

Major global efforts to address the COVID-19 pandemic have led to the urgent development of mRNA vaccines (BNT162b2 (Pfizer/BioNTech), mRNA-1273 (Moderna)), demonstrating their high efficiency in Phase 3 clinical trials. However, these trials incorporated a limited number (0.1%) of patients with hematological malignancies [8,9]. Moreover, none of these patients had received an HSCT. Notably, such patients are known to have an attenuated immune response to vaccines, especially in the early months after transplantation, as evidenced with the influenza vaccination [10,11]. The aforementioned factors make critical the assessment of immunogenicity, safety, and efficacy of anti-COVID-19 vaccines in this patient population. Earlier published data on the serological response to SARS-CoV-2 vaccines after allogeneic HSCT reported a wide range of response rates, varying from 37% to 96%, at 7–28 days after the second vaccine dose [12,13,14,15,16,17,18,19,20,21]. However, the information on the dynamics of anti SARS-CoV-2 antibody titers and response to the third vaccine dose in this patient population is currently limited [14,22,23,24].

Recent studies have shown a significant decrease in the humoral response and clinical efficacy of the BNT162b2 vaccine at 6 months post-vaccination in the general population [22,23]. These adverse dynamics might be even more pronounced in immunocompromised patients. 

The goals of the present study have been to evaluate the longitudinal immunological response and clinical efficacy of the BNT162b2 mRNA vaccine in allogeneic HSCT recipients and to determine predictive factors for response as well as the potential impact of a booster vaccine dose on the immunity in this vulnerable patient population. 

## 2. Patients and Methods

This multi-center prospective cohort study, conducted in three HSCT centers in Israel, included patients ≥18 years of age. Patients infected with COVID-19 prior to their second vaccination were considered ineligible for this study. Following authorization of the BNT162b2 mRNA vaccine (Pfizer/BioNtech) by the Israel Ministry of Health, a national mass adult vaccination program was launched on 19 December 2020. This also included high-risk patients, such as recipients of allogeneic HSCT. Similar to the recommendations for the general population, patients were assigned to receive two vaccine doses 21 days apart, which was in line with the guidelines issued by the European Society for Blood and Marrow Transplantation [24]. Based on these guidelines, vaccine administration was recommended from 3 months after the transplantation to patients with no acute graft-versus-host disease (GVHD) grades 3–4 and at least 6 months from the last anti-CD20 treatment. On 18 July 2021, the use of the third BNT162b2 dose was approved, and HSCT recipients with a 5-month lapse since their second vaccine dose were advised to receive the third dose. 

The study was approved by the Institutional Review Boards of the participating centers (approvals #0872-20-RMC, 0133-21-TMC, and 0211-21-HMO). All the patients signed the informed consent form. Patient demographic and medical data were retrieved from their electronic medical records. Blood samples for serology were taken 1, 3, and 6 months after the second vaccine dose and at 1 and 3 months after the third vaccination. Cellular response was evaluated 3 to 6 months after the second dose and compared to that of healthy controls.

### 2.1. Serology Testing for Anti-S SARS-CoV-2 Antibodies

Antibody (Ab) levels were measured using the SARS-CoV-2 IgG II Quant (Abbott^©^, Chicago, IL, USA) assay. The test was performed according to the manufacturer’s instructions for a quantitative measurement of IgG antibodies against the spike protein of SARS-CoV-2. Anti-S Ab levels ≥ 150 AU/mL were regarded as a positive response, while levels < 50 AU/mL were defined as a negative response. For the study analysis, an indeterminate response (an Ab level between 50 and 149 AU/mL) was considered a negative response. 

Patients were considered as responders, if they had a positive response (Ab levels ≥ 150 AU/mL) at one month after second vaccination. Those patients whose one-month test was not available, but their antibody levels were ≥150 AU/mL at 3 or 6 months after the second vaccine dose were also considered responders, assuming that these patients must have had higher levels at earlier time points.

### 2.2. Cellular Response

The T-cell response was evaluated with the Act-T4 Cell kit (Cytognos, Salamanca, Spain) according to the manufacturer’s instructions. In brief, 250 μL of sodium heparin anticoagulated whole blood was mixed with 250 μL of RPMI-1640 (Sigma-Aldrich, St. Louis, MO, USA). Three samples were incubated for each patient: a negative control sample without any stimulation; a positive control sample mixed with phytohemagglutinin (PHA; 10 μg/mL; Sigma-Aldrich, St. Louis, MO, USA); and a test sample co-cultured with spike glycoprotein (20 μg/mL; PepTivator SARS-CoV-2 Prot_S Complete—research grade; Miltenyi Biotec, Bergisch Gladbach, Germany). Cultures were incubated at 37 °C for 48 h in a humidified atmosphere of 5% CO_2_. 

Following incubation, 100 μL of each culture was transferred into a FACS tube and combined with an antibody mixture, including anti-CD3-PerCP-Cy5.5, anti-CD4-FITC, anti-CD25-APC, anti-CD134-PE (all four supplied with the Act-T4 Cell Kit; Cytognos, Salamanca, Spain), anti-CD27-BV421 clone M-T271 (Biolegend, San Diego, CA, USA), and anti-CD45RO-PE-Cy7 clone UCHL1 (BD Biosciences, NJ, USA). One mL of erythrocyte-lyse-no-wash solution was added to each tube, mixed with the tube contents and incubated for 10 min at the room temperature, protected from light. Stained cells were then assessed using BD FACSLyric^TM^ (BD Biosciences, NJ, USA) flow cytometer. Data were analyzed with Infinicyt ver. 2.5 A (Cytognos, Salamanca, Spain). CD3 T cells, CD4 T cells, and their subpopulations were evaluated for abundance and response according to the expression levels of the activation markers CD134 and CD25 (Figure S1 (Supplementary Materials)) [25,26]. 

### 2.3. Statistical Analysis

Categorical variables were analyzed using the Fisher exact test. The Kolmogorov–Smirnov test was employed to determine the normal distribution for continuous variables. Accordingly, the Mann–Whitney U test or *t*-test were used to analyze continuous variables. For all the methods, a *p*-value < 0.05 was considered statistically significant. IBM SPSS Statistics, version 27 was used to perform all the analyses.

## 3. Results

### 3.1. Patients

Seventy-nine patients vaccinated with two doses of BNT162b2 post-transplant were enrolled. Seventy-seven individuals, for whom all the relevant data were available, were included in the final analysis. The median age of the study participants was 55 years (range 20–74) and 53% of the cohort were females. The majority of patients (*n* = 55, 71%) were diagnosed with acute myeloid leukemia or high-risk myelodysplastic syndrome. Fifty-six percent underwent HSCT from a matched related donor, seventy-five percent were in complete remission (CR) prior to transplant, and all were in CR at the time of vaccination. More than half of the patients received a myeloablative conditioning (MAC) regimen, and antithymocytic globulin (5 mg/kg/day for 3 days) was added to the treatment in 56% of the patients. Chronic GVHD was considered active among 44% of the patients at the time of vaccinations and steroids (up to a maximal dose of 30 mg/day) were used in 30% of patients (Table 1).

### 3.2. Serological Response to Vaccine

The majority of the patients (*n* = 43, 56%) received their BNT162b2 injections at >12 months post-transplant (late period), in 21 patients (27%) it was given between 6–12 months (intermediate period), and in 13 patients (17%)—between 3 and 6 months post-transplant (early period). A positive serological response was evident in 66.2% of the entire cohort (51/77 patients). Response rates for those vaccinated at later, intermediate, or early time points were 83.7%, 52.4%, and 30.7%, respectively. In a univariate analysis, responding patients were found to be younger (median age 48.7 years old vs. 57.1, *p* = 0.022), of female gender (65% vs. 31% in non-responding, *p* = 0.007), had a transplant from a matched related donor (53% vs. 16%, *p* = 0.003), were in CR prior to transplant (82% vs. 58%, *p* = 0.028), and were neither on steroid therapy at the time of vaccination nor exposed to anti-CD20 within the 12 months preceding the vaccine administration. Importantly, 70% of the responders received their vaccine more than one year after the transplant, while only 27% in the non-responding group were vaccinated within the same time period (*p* = 0.0001; Table 1). In a multivariate analysis, the female gender, a recent anti-CD20 therapy, and a longer time lapse between the HSCT and vaccination remained significantly associated with a defined serological response (Table S3).

### 3.3. Serological Response Dynamics

Forty-five of the fifty-one responding patients had the serology data available at 6 months after the second vaccine dose. At that time point, the antibody titers of 43 of these patients (95.5%) remained over 150 AU/mL. In two additional patients, the titers decreased to the levels below 150 AU/mL. 

**Figure 1:**
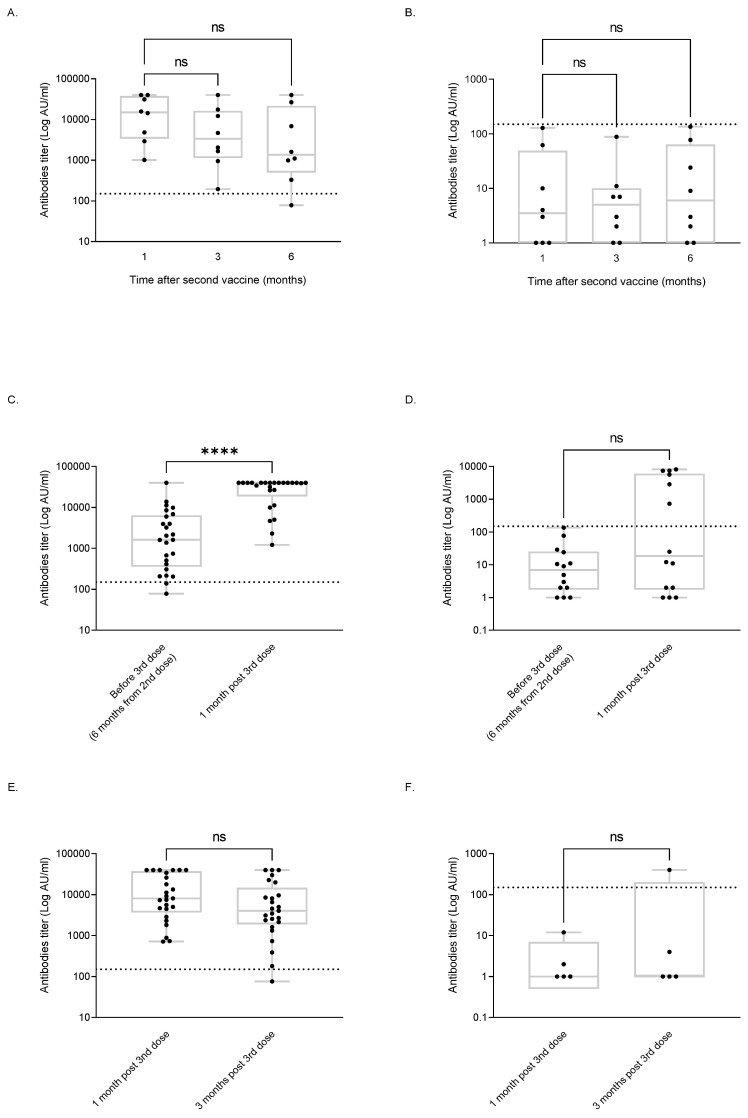
Antibody titer dynamics after the second and third vaccine doses in responders and non-responders. Antibody titer values are presented in Table S2. Titer dynamics after the second vaccine dose for responders, *n* = 8 (**A**) and non-responders, *n* = 8 (**B**). Antibody titers at 6 months from the second vaccination (before the third vaccine dose) and one month after the third vaccine dose in second dose responders, *n* = 25 (**C**) and non-responders, *n* = 14 (**D**). Antibody titer dynamics after the third vaccination in responders, *n* = 25 (**E**) and non-responders, *n* = 5 (**F**). **** *p* < 0.0001. ns—not significant.

However, the analysis of the findings on the 16 patients for whom the serology data at all the three first time points were available demonstrated that in 8 responders the median titer declined from 15,047 AU/mL [range 1010–40,000] at 1 month to 1355 AU/mL [range 78–40,000] at 6 months after the second vaccine dose (*p* = 0.13, Figure 1A, Table S2).

For a total of 39 patients, including 25 responders and 14 non-responders, serology data were available both at 6 months after the second vaccine dose and at 1 month after the third dose. In 23 of the responders, a titer above 150 AU/mL was observed at 6 months after the second dose and at 1 month after the third dose. Notably, the two responders who lost their response by 6 months after the second dose regained a titer of above 150 AU/mL after the third vaccination. Similarly, 6 of the 14 non-responders (43%) achieved a response with a titer above 150 AU/mL at 1 month after the third dose. The analysis of these data demonstrated that the overall number of responders to the BNT162b2 mRNA vaccine increased from 66% (51/77 patients) after two vaccinations to 79.5% (31/39 patients) after the administration of the third dose (Figure 1C,D).

Data on 30 patients who received three vaccine doses were evaluated for response dynamics at 1 and 3 months after the third vaccination. At 3 months after the third vaccination, 24 of the 25 (93%) responders to the third dose maintained their antibody titers ≥ 150 AU/mL, whereas in 4 of the 5 patients who failed to respond to the third vaccine dose, the antibody titers remained < 150 AU/mL. Surprisingly, one patient who did not respond either to the second dose or to the third one, as evaluated at 1 month following the latter vaccination, gained an antibody titer of 401 AU/mL at 3 months after receiving the third dose. Among the patients who responded to the third vaccine dose, the median antibody titer decreased from 8105 AU/mL [range, 715–40,000] at 1 month to 4026 AU/mL [range 76–40,000] at 3 months after this vaccination (*p* = 0.1, Figure 1E, Table S2).

### 3.4. Cellular Response

**Figure 2:**
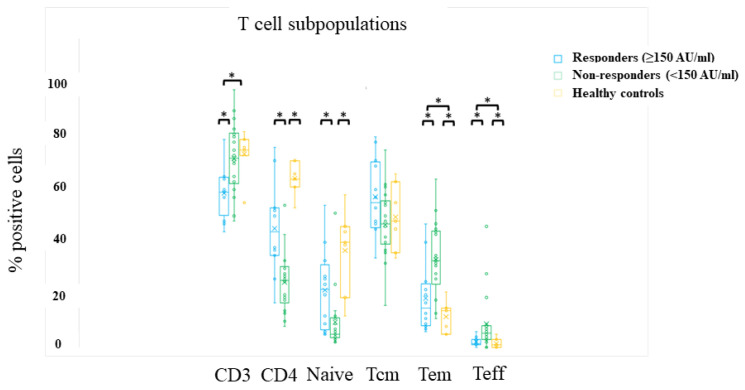
T-cell sub-populations. Comparing the abundance of T cells, T helper cells, and T helper subpopulations in vaccine responders (*n* = 12), non-responders (*n* = 18), and healthy controls (*n* = 7). Samples are from three to six months after the second vaccine dose. * denotes a significant difference between two compared populations (*p* < 0.05). Values are presented in Table S1. Tcm—T central memory; Tem—T effector memory; Teff—T effectors.

Cellular response was evaluated 3 and 6 months after the second vaccine dose. Patients’ cellular response was compared to that of seven healthy controls, who had a positive serologic response after the second vaccine dose. Flow cytometry analysis of baseline (pre-stimulation) abundance of T-cell subpopulations demonstrated significantly lower levels of CD3 T cells in responding patients compared to non-responders or healthy controls (Figure 2, Table S1), while CD4 and naïve T-cell levels were significantly lower in non-responding patients than in responders or healthy controls. 

**Figure 3:**
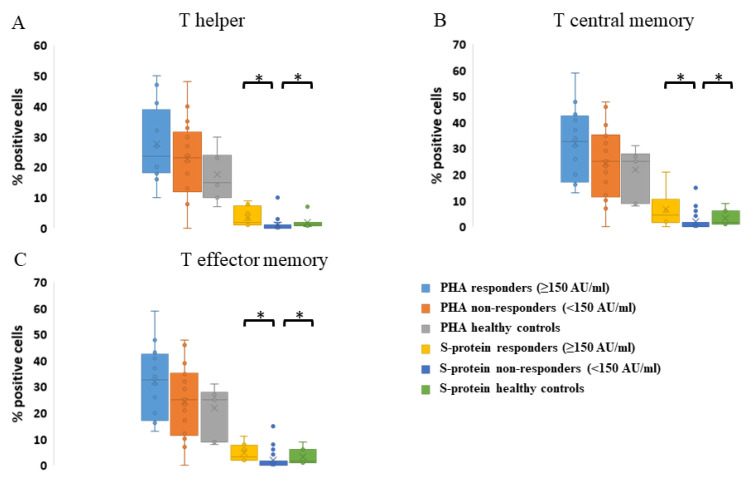
Cellular response. T-lymphocyte response to stimulation was evaluated by expression of the cellular activation markers CD134 and CD25 with flow cytometry. Samples were incubated for 48 h with PHA (positive control), no stimulation (negative control), or spike protein (test sample). Cellular response was evaluated in responding patients (antibody titers ≥ 150 AU/mL, *n* = 12), non-responding patients (antibody titers < 150 AU/mL, *n* = 18), and healthy controls (all had serological response to the vaccine, *n* = 7). (**A**) T-helper response to stimulation. (**B**) Central memory T-cell response to stimulation. (**C**) T-effector memory cell response to stimulation. There was no difference between the groups in response to PHA. Values are presented in Table S1. * *p* ≤ 0.001.

As for the levels of central memory CD4 T cells, they were similar in the three groups, but the abundance of T effector memory cells and T effectors was significantly higher in non-responders. All the three cell subpopulations, T helper, T central memory, and T effector memory, demonstrated a very strong response to PHA. Furthermore, all of them expressed significantly elevated levels of the activation markers in responding patients and healthy controls relative to non-responders (Figure 3).

The observed correlation between the cellular response to spike protein stimulation and the serological response further emphasizes the importance of the reconstitution of both humoral and cellular immunity after HSCT.

### 3.5. Clinical Effects of SARS-CoV-2 on HSCT Recipients

At a median follow-up of 284 days [interquartile range (IQR) 258–290] from the second vaccination, only one of the seventy-seven evaluated patients developed COVID-19 (at 5.5 months after the second vaccine dose). This patient had an antibody titer of 951 AU/mL at 3 months post the second vaccination and recovered completely after a mildly symptomatic disease. There was no difference in overall survival between responders and non-responders. Notably, the relapse rate of a hematological disease was 19% in non-responses and 2% in responders (*p* = 0.015). Chronic GVHD exacerbation or start by 30 days from the second vaccine dose occurred in 17% of the patients (7 out of 41 with available data), without a significant difference between the two groups. Among the 36 patients with a median follow-up of 742 days [IQR 697–764], there were 5 cases of COVID-19 infection at a median time of 360 [range 336–406] days after the second vaccine dose (median of 166 days post the third vaccination). All the five cases occurred between January and March 2022, during the dominance period of the Omicron BA.1 variant.

## 4. Discussion

The current study demonstrated the high immunogenicity of the SARS-CoV-2 BNT162b2 vaccine in recipients of allogeneic HSCT, which further increased if the vaccine was administered at a longer time lapse from the transplant. While the immunogenicity waned with time, it significantly recovered with the booster vaccine dose. Sufficient antibody titers (≥150 AU/mL) were achieved in 66.2% of the evaluated patients after the second vaccine dose. Furthermore, 43% of non-responding patients and all the patients who lost their response at 6 months after the second dose, achieved antibody levels ≥ 150 AU/mL after the third dose, which resulted in a total response rate of 79.5%. Notably, at 3 months following the third vaccination, as many as 93% of the responders maintained their antibody titers ≥ 150 AU/mL.

To the best of our knowledge, this is the first trial longitudinally assessing the immunological response to the BNT162b2 vaccine in allogeneic transplant recipients through a time period of up to 3 months from the administration of the third vaccine dose. 

The clinical efficacy of the BNT162b vaccine in healthy individuals was reported to be 95% after the second dose [9]. However, by 3 months post-vaccination, the antibody titers declined, which was particularly prominent in individuals aged > 60 years, males, and in those with underlying conditions [27]. An average titer decrease reached 70% by 6 months from the second vaccination [28]. This translated into a significant reduction in the vaccine efficacy, which was evident in all age groups [29]. Moreover, the protection against SARS-CoV-2 variants B.1.351 (beta) and B.1.617.2 (delta) dropped to 20% 5–7 months after the second dose [30]. Likewise, in a cohort of 106 allogeneic HSCT recipients, a median decline of 20% per month in the antibody titers was documented between 2 and 6 months post the second vaccination [21]. A trend of titer decline was also observed in this study. 

The gradual waning immunity in the general population was associated with an increase in COVID-19 prevalence and led to the implementation of a third vaccine dose, administered 5 months after the second one. This policy resulted in a significant elevation in antibody titers and improved clinical efficacy of vaccination [23,31]. Several recent studies reporting findings on a total of 515 adult allogeneic HSCT recipients demonstrated the efficacy of a third anti SARS-CoV-2 vaccination evaluated either by an increase in antibody titer levels or in the number of responders [14,18,24,32]. Le Bourgeois et al. reported a response rate of only 50% in patients receiving two vaccine doses compared to a response rate of 81% in another group of patients vaccinated with the third dose [33]. In a similar vein, Canti et al. demonstrated a significant increase in antibody titer levels prior to (median of 152 BAU/mL) and one month after the third vaccine dose (median of 2955 BAU/mL) in 38 patients who had undergone an allogeneic HSCT [12]. A study from the University of Kansas Medical Center reported data on 100 patients, with a responder rate of 65.8% and 72.4% at 1 and 6 months after the second vaccine dose, respectively, further increasing this rate to 90.6% at 1 month after the third dose [32]. Similarly, Kimura et al. evaluated immunogenicity in a cohort of 95 patients, demonstrating a responder rate of 51% at 1 month after the second dose that increased to 81.2% at 1 month after the third vaccination [34]. 

Furthermore, the third vaccine was shown to be efficient in non-responders to the second vaccine dose. The study by Redjoul et al. reported the effects of the third vaccination on 42 such patients, 48% of whom turned to responders following the third vaccination, as confirmed by a significant increase in the antibody titers (from a median of 737 AU/mL prior to the booster to a median of 11,099 AU/mL by one month post-vaccine injection) [14]. Along the same lines, a multicenter French study, assessing 181 patients receiving the third vaccine dose, found that 42% of those with a weak or no response after the second dose became good responders following the third vaccination [14,35]. Similarly, in the current study, 43% of non-responding patients gained a response with an antibody titer ≥ 150 AU/mL following the third vaccine dose.

In the present study, at a median follow-up of over 9 months since the second vaccination, only one patient in the responder group and none among non-responders developed the COVID-19 disease. This might be attributed to the meticulous compliance of this population with the national recommendations aimed to ensure protection against COVID-19 (e.g., wearing a face mask, social distancing, avoiding crowds, and practicing hand hygiene). In four other studies evaluating at an early time the clinical efficacy of anti-SARS-CoV-2 vaccines in 200 allogeneic HSCT recipients, only 1 case of COVID-19 was reported [12,13,14,21]. Altogether, this resulted in an early time total infection rate of 0.7% (2/277) in the five studies. In a large registry study reported by Mittelman et al. of 5107 patients on active hematological therapy (currently treated and within 6 months from the latest treatment) who were vaccinated with two BNT162b2 vaccine doses, 37 patients acquired COVID-19 (infection rate 0.7%). Furthermore, only 4 of 1196 post-HSCT patients included in that analysis had COVID-19 disease (infection rate 0.3%). While these rates seemed low, the infection rate in matched controls from this registry were lower (0.2% and 0.1%, respectively) [36], which emphasizes the vulnerability of this patient population despite the beneficial contribution of vaccination to their protection. 

In the current study, at a median follow-up of 1 year after the third vaccination, 14% of patients (5 out of 36) were infected with COVID-19, presumably the Omicron BA.1 variant which was the epidemiologically dominant variant at the time the infections occurred (January–March 2022). This rate of infection is similar to the findings of other studies reporting an infection rate of 11–16% in allogeneic HSCT recipients, all with a mild disease course [34,37]. All these data are consistent with the known decreased efficacy of the BNT2b2 vaccine against the Omicron variant [38]. 

The present study identified the time from transplant to vaccine administration to be strongly associated with humoral response. Other factors contributing to a sufficient response included: younger age, female gender, no use of steroids, no exposure to anti-CD20 therapy within the preceding 12 months, CR during HSCT, and a transplant from a matched related donor. While all similar studies uniformly recognized the significant impact of time from transplant to vaccination, there was less consistency with regard to a potential effect of other factors, such as immunosuppressive treatment, high-grade chronic GVHD, and reduced intensity conditioning [12,13,14,15,16,17,18,19,20,21]. Notably, none of the studies reported an association between recent use of anti-thymocyte globulin (ATG) and attenuated humoral response in HSCT patients. 

Reconstitution of cellular immunity after HSCT is critical for the induction of an effective humoral response. This may take months or even years [39] and supports the current observation of higher efficacy of the anti-COVID-19 vaccination when given at a longer time span from transplant, compared to that received early post-HSCT. Furthermore, this may partially explain why, in the present study, the booster dose administered at least 8 months post-transplant was significantly more effective than the second vaccine dose. T cells and particularly CD4 T helper cells have a cardinal role in sustaining immunity after vaccination with BNT162b2. Their specific activation mainly occurs after the first vaccine dose and significantly increases after the second one. In healthy individuals, the vaccine-induced T cells are of the central memory (Tcm) and effector memory (Tem) phenotype [40]. In the current study, while the baseline frequency of Tcm was similar in healthy controls, responders, and non-responders and Tem frequency was even higher in non-responders, activation of these cells with the spike protein was significantly more prominent in the two former groups. The Tcm and Tem reaction to the spike protein stimulation reflects true cellular memory induced in the responder group rather than the abundant presence of these cells.

Although other studies reported a correlation of elevated frequency of naïve CD4 T cells, B-cells, NK cells, and CD3 T cells with sufficient humoral response to vaccine in the post-transplant setting [12,13,14,18], our data suggest that the immunity of allogeneic HSCT recipients after BNT162b vaccination depends on the potency of T helper lymphocytes rather than on their frequency.

This study has several limitations. The relatively small cohort size and limited availability of serological and cellular data for all patients at the evaluated time points, inter-center heterogeneity of other transplant-related factors that could potentially affect the immune response (non-myeloablative conditioning, etc.), and the fact that this trial evaluated only the BNT162b2 mRNA vaccine, could somewhat reduce the applicability of the obtained results. However, the findings of the present study are in line with those of other trials, which contributes to their relevance. In this study, the definition of an adequate response was based on the antibody titer cutoff set at 150 AU/mL, while others used different assays with different cutoff values to define sufficient response. This may account for differences between study findings, that are mainly related to the factors associated with sufficient immunity. Furthermore, the vaccination efficacy observed in this study was not compared to historical matched controls, i.e., allogeneic HSCT recipients prior to vaccine availability. This would have given a more accurate estimation of vaccine effectiveness in these patients. Additionally, the detected antibodies were not measured for their ability to act as neutralizers, but this was rather inferred based on the clinical data of the COVID-19 cases, that were few in numbers and characterized by a mild course. 

## 5. Conclusions

The BNT162b2 vaccine administered to allogeneic HSCT recipients, starting at least 3 months post-transplant, is effective in eliciting sufficient immunogenic response. This response is likely enhanced if the vaccine is administered at a longer interval from the transplant and is significantly improved after the booster dose. A long-term follow-up of vaccine immunogenicity and efficacy is imperative to establish health recommendations specifically targeting this highly vulnerable population.

## Figures and Tables

**Table 1:** Patient characteristics and outcomes.

Evaluated Parameters	All Patients (%)	Responders (Ab Titer ≥ 150 AU/mL); *n* = 51 (%)	Non-Responders (Ab Titer < 150 AU/mL); *n* = 26 (%)	*p* Value
Age, years; mean ± SD	55 ± 15.4	48.7 ± 16.1	57.1 ± 12.2	0.022
Female gender	41 (53)	33 (65)	8 (31)	0.007
Diagnosis				0.097
AML	43 (56)	31 (61)	12 (46)	
ALL	11 (14)	8 (16)	3 (12)	
MDS	12 (15.5)	6 (12)	6 (23)	
MPN	5 (6.5)	1 (2)	4 (15)	
LY	6 (8)	5 (10)	1 (4)	
HSCT-CI; median [IQR]	1.5 [0–3]	2.5 [0.25–3.75]	1 [0–2]	0.18
Number of pre-transplant lines of therapy				0.15
1	45 (83.3)	22 (76)	23 (92)	
≥2	9 (16)	7 (24)	2 (8)	
Donor				0.003
MRD	31 (40.8)	27 (53)	4 (16)	
MUD	38 (50)	22 (43)	16 (64)	
Haploidentical	7 (9.2)	2 (4)	5 (20)	
Conditioning intensity				0.73
MAC	43 (55.8)	29 (57)	14 (54)	
RTC	19 (24.6)	11 (22)	8 (31)	
RIC	14 (18.2)	10 (20)	4 (15)	
NMA	1 (1.4)	1 (2)	0	
* Disease status at transplant				0.028
CR	57 (74)	42 (82)	15 (58)	
Other	20 (26)	9 (18)	11 (42)	
ATG	43 (56)	26 (51)	17 (65)	0.33
TBI	12 (15.6)	8 (16)	4 (15)	>0.99
GVHD prophylaxis				0.56
CSA MTX	48 (62.3)	30 (59)	18 (69)	
CSA MMF	28 (36.4)	20 (39)	8 (31)	
CSA only	1 (1.3)	1 (2)	0	
PTCy	5 (6.5)	1 (2)	5 (19)	0.084
Anti-CD20 therapy within previous 12 months	4 (5.2)	1 (2)	3 (11.5)	0.038
Time from transplant to vaccine administration				<0.0001
Early, <6 months	13 (16.9)	4 (8)	9 (35)	
Intermediate, 6–12 months	21 (27.3)	11 (22)	10 (38)	
Late, >12 months	43 (55.8)	36 (70)	7 (27)	
Chronic GVHD at time of vaccination	34 (44.1)	21 (41)	13 (50)	0.48
Steroid treatment at time of vaccination	23 (29.9)	11 (22)	12 (46)	0.036
Stem cell donor age, years, mean ± SD (*n* = 47)	33.5 ± 14.9	37.4 ± 16.3	30.04 ± 13.4	0.095
Female donor (*n* = 47)	11 (23.4)	5 (22)	6 (25)	0.99<
% donor chimerism at 1 month post-HSCT, mean ± SD (*n* = 47)	99.3 ± 1.6	99.04 ± 1.85; *n* = 23	99.5 ± 1.56; *n* = 24	0.32
GVHD exacerbation or onset at 1 month post-vaccination (*n* = 41)	7 (17)	4 (22)	3 (13)	0.68
Disease relapse	5 (6.5)	1 (2)	5 (19)	0.015
Alive 7 months after second vaccine dose (*n* = 41)	37 (90)	17 (94); *n* = 18	20 (87); *n* = 23	0.62

* CR—complete response, including CR1, CR2, CR3; other—includes partial response, relapse, refractory disease. When data were not available for all the patients in the cohort, the number of patients with data was stated (*n*); AML—acute myeloid leukemia; ALL—acute lymphocytic leukemia; MDS—myelodysplastic syndrome; MPN—myeloproliferative neoplasm; LY—lymphoma; IQR—interquartile range; HSCT-CI-hematopoietic stem cell transplantation comorbidity index; MRD—matched related donor; MUD—matched unrelated donor; MAC—myeloablative conditioning; RTC—reduced toxicity conditioning; RIC—reduced intensity conditioning; NMA—non-myeloablative; ATG—anti-thymocyte globulin; TBI—total body irradiation; CSA—cyclosporine; MTX—methotrexate; MMF—mofetil mycophenolate; PTCy—post-transplant cyclophosphamide; GVHD—graft-versus-host disease.

## Data Availability

The data that support the findings of this study are available from the corresponding author upon reasonable request.

## Supplementary Materials

The following supporting information can be downloaded at: https://www.mdpi.com/article/10.3390/vaccines11040775/s1, Figure S1. Cellular response gating technique and analysis; Table S1. Cellular response values; Table S2. Antibody titer values after the second and third vaccine doses in responders and non-responders for the entire cohort; Table S3. Multivariate analysis for response to the second vaccine dose.
