# Supplementary material for: Third BNT162b2 mRNA SARS-CoV-2 Vaccine Dose Significantly Enhances Immunogenicity in Recipients of Allogeneic Hematopoietic Stem Cell Transplantation
Source: Vaccines (Basel). 2023 Mar 31;11(4):775. doi: 10.3390/vaccines11040775 (PMC10145595; doi:10.3390/vaccines11040775)
Supplement: Supplementary file 1 [file vaccines-11-00775-s001.zip › vaccines-2246788-supplementary.pdf]

Figure S1. Cellular response gating technique and analysis

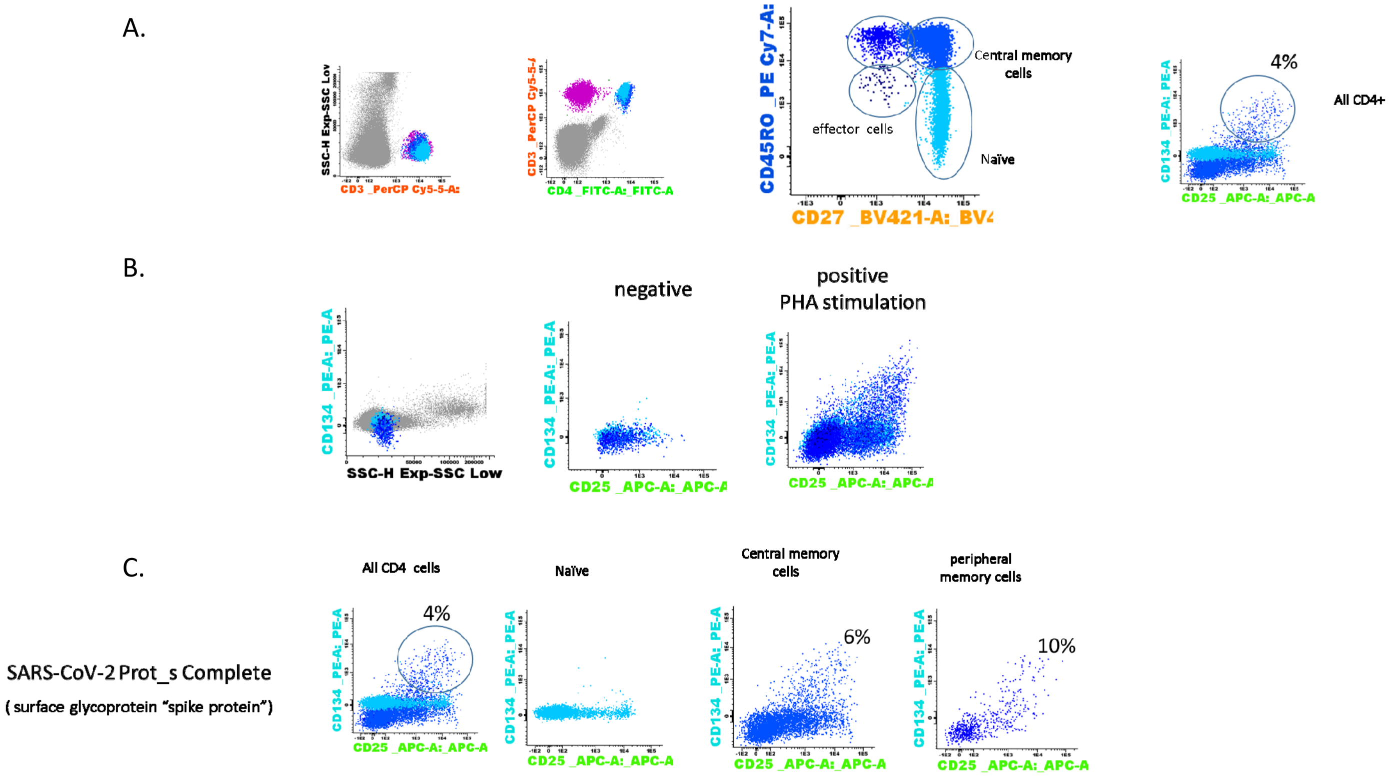

A) CD3 T lymphocytes were gated. CD4 T helper lymphocytes were gated out of the CD3 population. CD4 sub-populations were gated according to their expression of CD45RO and CD27: Naïve cells (CD45RO-, CD27+), T central memory cells (CD45RO+, CD27+), T effector memory (CD45RO+, CD27-), T effectors (CD45RO-, CD27-).[41] B-C) To find the frequency of activated cells to stimulation, each subpopulation was gated for CD134+ and CD25+ double expresser cells.[42] B) stimulation with PHA. C) stimulation with the spike-protein.

Table S1. Cellular response values

	**Responders (≥150 AU/ml); n=12**	**Non-responders (<150 AU/ml) ; n=18**	**Healthy controls; n=7**	**P value**
**T cells (CD3+)**	59 [50.25-64.75]	72 [62.25-81.5]	75 [73-79]	**P1=0.007**<br>**P2=0.013**<br>P3=0.071
**T helper (CD4+)**	44 [35-53]	25.5 [17-30.75]	64 [61-71]	**P=0.002**<br>P2=0.078<br>**P3<0.001**
**% Activated cells (CD134+, CD25+)**				
**Negative control**	0	0	0	0
**Positive control (PHA)**	23.5 [18.25-38.75]	23 [12-31.5]	15 [10-24]	P=0.46<br>P=0.25
**Test samples (s-protein)**	2 [1.13-7.25]	0 [0-0.95]	1.3 [0.7-2]	**P=0.001**<br>P2=0.29<br>**P3<0.001**
**Naïve CD4 T cells**	22 [6.75-31.50]	5 [3.75-11.25]	40 [19-46]	**P=0.007**<br>P2=0.15<br>**P3<0.001**
**% Activated cells (CD134+, CD25+)**				
**Negative control**	0	0	0	0
**Positive control (PHA)**	0	0	4 [0-5]	**P2=0.017**<br>**P3=0.001**
**Test samples (s-protein)**	0	0	0	0
**Central memory CD4 T cells**	55 [45.5-70.5]	47 [39.25-55.75]	48 [36-63]	P1=0.09<br>P2=0.22
**% Activated cells (CD134+, CD25+)**				
**Negative control**	0	0	0	0
**Positive control (PHA)**	32.5 [17-42.50]	25 [11.5-35.25]	25 [9-28]	P1=0.20<br>P2=0.25
**Test samples (s-protein)**	3 [2-7.75]	0 [0-1.53]	1.5 [1-6]	**P=0.001**<br>P2=0.30<br>**P3<0.001**
**Effector memory CD4 T cells**	15 [8.25-24.25]	33 [24-44.25]	14 [5-15]	**P=0.008**<br>**P2=0.010**<br>**P3=0.001**
**% Activated cells (CD134+, CD25+)**				
**Negative control**	0	0	0	0
**Positive control (PHA)**	28 [23.75-55.5]	26.5 [13.25-31.25]	22 [14-37]	P=0.13<br>P=0.23
**Test samples (s-protein)**	3 [1-9.75]	0 [0-0.25]	3 [2-10]	**P=0.001**<br>**P2=0.58**<br>**P3<0.001**
**Effector CD4 T cells**	1.5 [1-3]	5.5 [3-8.25]	1 [0-3]	**P=0.004**<br>**P2=0.007**<br>**P3=0.002**
**% Activated cells (CD134+, CD25+)**				
**Negative control**	0	0	0	
**Positive control (PHA)**	0	0	0	
**Test samples (s-protein)**	0	0	0	

Frequencies are presented as percent of positive cells. Median and range [..] are presented. P1 = responders vs. non-responders, P2 = responders vs. healthy controls, P3=non-responders vs. healthy controls.

Table S2. Antibody titer values after the second vaccine dose in responders and non-responders for the entire cohort

**Antibody titer (AU/ml) after vaccine second dose, median [range]**	**Responders* (≥150 AU/ml)**	**Non-responders* (<150 AU/ml)**	**P value**
**1 month**	2917 [401-40000], N=14	10 [0-148], N=17	<0.0001
**3 months**	1628.5 [33-40000], N=17	5 [0-133], N=20	<0.0001
**6 months**	1612.5 [78-40000], N=45	3 [0-135], N=17	<0.0001
**1 month post 3rd dose**	40000 [881-40000], N=30	715 [0-17879], N=19	<0.0001
**3 month post 3rd dose**	8524 [76-40000], N=15	1030 [0-4560], N=16	<0.0001

Response to the second vaccine dose.

N - Number of patients with available data.

Table S3. Multivariate analysis for response to the second vaccine dose

**Response (≥150AU/ml)**			
**Predictors**	Odds ratio	95% CI	P value
**Gender [female]**	8.551	1.931 - 53.65	**0.0095**
**Age**	0.9721	0.9159 - 1.026	0.3176
**Donor [haplo]**	0.05442	0.0005306 - 2.546	0.1650
**Donor [MRD]**	3.901	0.3243 - 70.24	0.3124
**Disease status [CR]**	1.599	0.2946 - 8.672	0.5798
**Anti CD20 [yes]**	0.03284	0.0006897 - 0.5525	**0.0359**
**ATG [no]**	0.8126	0.05485 - 11.84	0.8761
**Vaccine timing [int]**	2.597	0.4056 - 19.06	0.3212
**Vaccine timing [late]**	11.59	1.771 - 104.1	**0.0158**
**Steroids Rx at vaccine [no]**	3.610	0.7187 - 20.17	0.1220

Haplo – haploidentical donor, MRD – matched related donor, CR – complete remission, int - intermediate time period after transplant (>6 months and < 12 months), late - intermediate time period after transplant (> 12 months). Multiple logistic regression.
